# Role of Different Forms and Intensities of Physical Activity in Prevention of Dementia

**DOI:** 10.1192/j.eurpsy.2024.432

**Published:** 2024-08-27

**Authors:** I. Singh, R. Chandra

**Affiliations:** ^1^Mental Health, Central West Hospital & Health Service, Longreach; ^2^Grampians Health Services- Aged Persons Mental Health Services, Ballarat, VIC, Australia

## Abstract

**Introduction:**

Dementia is one of the greatest health challenges worldwide. According to the World Health Organisation (WHO) factsheet, currently more than 55 million people worldwide have dementia, with over 60% living in low- and middle-income countries. Every year, there are nearly 10 million new cases of dementia. WHO Global Status Report 2021 estimated growth of 139 million people with dementia by 2050. The estimated total global cost of dementia is likely to surpass US$ 2.8 trillion by 2030. As one grows older, the risk of developing dementia, particularly Alzheimer’s Disease, progressively increases. The Lancet Commission Reports 2017 and 2020 on dementia prevention, intervention, and care identified 12 modifiable risk factors, including physical inactivity, obesity, midlife hypertension, and diabetes. Addressing these lifestyle factors may significantly reduce the risk of dementia and its progression. While no curative or disease-modifying treatment is available for dementia at his stage, addressing modifiable risk factors may have a preventive role in reducing the risk of dementia.

**Objectives:**

The objective of the literature review is to explore current evidence on Physical Activity (PA) in reducing the risk of developing dementia and its progression. The focus is also to see the association of different forms and intensities of PA and their intensities, including aerobic, strength-based, and leisure, with the risk and progression of dementia.

**Methods:**

Narrative review

**Results:**

Results from the reviewed studies showed that PA was found to be associated with a reduced risk of dementia, particularly Alzheimer’s Disease. Studies comparing different intensities of PA indicate though all levels of PA decrease the risk of dementia, there is a linear relationship between the higher intensity PA and the increased beneficial effect in terms of reduced risk of dementia. Leisure-time PA also has a protective role against dementia in longitudinal studies. There is more consistent evidence in favour of aerobic PA; however, it has a ceiling effect. The combination of aerobic and strength-based experience provides optimum beneficial effects. The elderly population who started physical activity in their 80s experienced the beneficial effects of PA in reducing the risk of dementia. There is mixed evidence of the protective effect of PA on the population who have already developed cognitive impairment or have genetic vulnerabilities. The author will also include the results of any relevant study published by 31 March 2024.

**Conclusions:**

The details of the literature results and conclusions will be discussed at the conference.

**Disclosure of Interest:**

I. Singh Consultant of: The author declare that the review was conducted in the absence of any commercial or financial relationships that could be construed as a potential conflict of interest., R. Chandra: None Declared

